# Safety and Efficacy of Biodegradable Drug-Eluting vs. Bare Metal Stents: A Meta-Analysis from Randomized Trials

**DOI:** 10.1371/journal.pone.0099648

**Published:** 2014-06-19

**Authors:** Yangguang Yin, Yao Zhang, Xiaohui Zhao

**Affiliations:** 1 Cardiovascular Disease Research Center, Xinqiao Hospital, Third Military Medical University, Chongqing, China; 2 The Evidence Based Medicine and Clinic Epidemiology Center, Third Military Medical University, Chongqing, China; S.G. Battista Hospital, Italy

## Abstract

**Background:**

Biodegradable polymeric coatings have been proposed as a promising strategy to enhance biocompatibility and improve the delayed healing in the vessel. However, the efficacy and safety of biodegradable polymer drug-eluting stents (BP-DES) vs. bare metal stents (BMS) are unknown. The aim of this study was to perform a meta-analysis of randomized controlled trials (RCTs) comparing the outcomes of BP-DES vs. BMS.

**Methods and Results:**

PubMed, Embase, and Cochrane Central Register of Controlled Trials (CENTRAL) were searched for randomized clinical trials, until December 2013, that compared any of approved BP-DES and BMS. Efficacy endpoints were target-vessel revascularization (TVR), target-lesion revascularization (TLR) and in-stent late loss (ISLL). Safety endpoints were death, myocardial infarction (MI), definite stent thrombosis (DST). The meta-analysis included 7 RCTs with 2,409 patients. As compared with BMS, there was a significantly reduced TVR (OR [95% CI] = 0.37 [0.28–0.50]), ISLL (OR [95% CI] = −0.41 [−0.48–0.34]) and TLR (OR [95% CI] = 0.38 [0.27–0.52]) in BP-DES patients. However, there were no difference for safety outcomes between BP-DES and BMS.

**Conclusions:**

BP-DES is more effective in reducing ISLL, TVR and TLR, as safe as standard BMS with regard to death, ST and MI. Further large RCTs with long-term follow-up are warranted to better define the relative merits of BP-DES.

## Introduction

The development of bare-metal stents (BMS) represents a considerable advance over balloon angioplasty in preventing restenosis by attenuating early arterial recoil and contraction. However, 15% to 20% of patients required ≥1 repeat revascularization procedure within the 6 to 12 months after BMS implantation [1]. Polymer based drug-eluting stents (DES) are currently widely used to reduce restenosis and the need for repeat revascularization, representing a major advance for percutaneous coronary intervention (PCI). [2] However, well publicized concerns raises with the long-term safety of stent thrombosis (ST) [3].

At present, great efforts have been prompted to develop alternative stents with biodegradable polymers (BP) for drug delivery, which degrade over time, and therefore hope to provide comparable long term safety to BMS while maintaining the early antirestenosis of DES. Previous studies have shown biodegradable polymer drug-eluting stents (BP-DES) is a safe and efficacious alternative to conventional durable polymer DES [4], [5], [6]. However, uncertainty exists regarding the relative performance of BP-DES vs. BMS.

## Methods

Established methods [7] were used in compliance with the PRISMA statement for reporting systematic reviews and meta-analyses in health care interventions [8].

### Search Strategy

We searched Embase, PubMed, and Cochrane Central Register of Controlled Trials (CENTRAL) for studies on BP-DES until December 2013. The search strategy was formulated as the AND-combination of terms 1) Polymer 2) Stent, in Randomized controlled trials (RCTs). There was no language restriction for the search.

References of meta-analyses, review articles, and original studies identified by the electronic searches were manually checked for additional trials. For studies that did not report outcomes of interest, efforts to contact authors were performed to obtain further details. Internet-based sources of information on the results of clinical trials in cardiology www.theheart.org, www.cardiosource.com/clinicaltrials, www.clinicaltrialresults.com, and www.tctmd.com) were also searched. In addition, we searched conference abstracts of the following societies: American College of Cardiology, Transcatheter Cardiovascular Therapeutics, American Heart Association, European Society of Cardiology, Society of Cardiovascular Angiography and Intervention and Euro-PCR.

### Selection Criteria

Inclusion criteria were: 1. Human studies related to PCI. 2. RCTs. 3. BMS as control. Exclusion criteria were: 1. Non-RCT; 2. Sub-study of the RCT. Two authors (Yangguang Yin and Yao Zhang) independently assessed trial bias risk and extracted data.

### Data Extraction and Synthesis

Efficacy outcomes were target-lesion revascularization (TLR), target-vessel revascularization (TVR) and in-stent late loss (ISLL). Safety outcomes were death, myocardial infarction (MI) and stent thrombosis (ST). Stent thrombosis was defined as Academic Research Consortium (ARC) [9]. TLR or TVR defined as any revascularization procedure involving the target lesion or vessel owing to luminal re-narrowing in the presence of symptoms or objective signs of ischemia, respectively.

### Quality Assessment

The CONSORT 2010 Statement, as a standard for the quality control assessment, was applied to evaluate the quality of the studies included. For each evaluation criterion of the CONSORT 2010 Statement, we assigned ‘Adequate’, ‘Not Adequate’, or ‘Unclear’ to evaluate the quality of the 7 RCTs included. The following criteria were used: Adequate indicated low bias and completely fulfilled quality standards with the least bias; Unclear indicated a lack of information or bias uncertainty; and Not Adequate was assigned if the criteria were completely unfulfilled or there was a high likelihood of bias. If a trial completely fulfilled at least six quality standards of the 10 inclusion/exclusion criteria, it was considered to be of high quality. Two reviewers (Yangguang Yin and Yao Zhang) independently evaluated and cross-checked the quality and assessed the bias of the literatures.

### Statistical Methods

All statistical tests were performed using the Cochrane Collaboration’s Revman5.2.6.

The chi-square test was used to examine differences in categorical variables, such as the frequencies, A P value<.05 was considered statistically significant. Summary estimate includes odds ratio (OR), Standard Mean Difference, (SMD) and its 95% confidence intervals (CI) were used as summary statistics in forest plot.

Heterogeneity among studies was determined by the Chi-square-based Q test and the I^2^ statistics. A p value less than 0.05 for the Q test together with an I^2^ value greater than 50% was considered a measure of severe heterogeneity. Therefore, the pooled OR estimate of each study was calculated using the fixed-effect model (the Mantel–Haenszel method); otherwise, the random-effects model (the DerSimonian and Laird method) was used. The Potential publication bias for each of the pooled study groups was assessed with a funnel plot. A two-tailed test was used to assess the funnel plot asymmetry; the significance was set at p<.05 level.

## Results

### Study Selection

**Figure 1:**
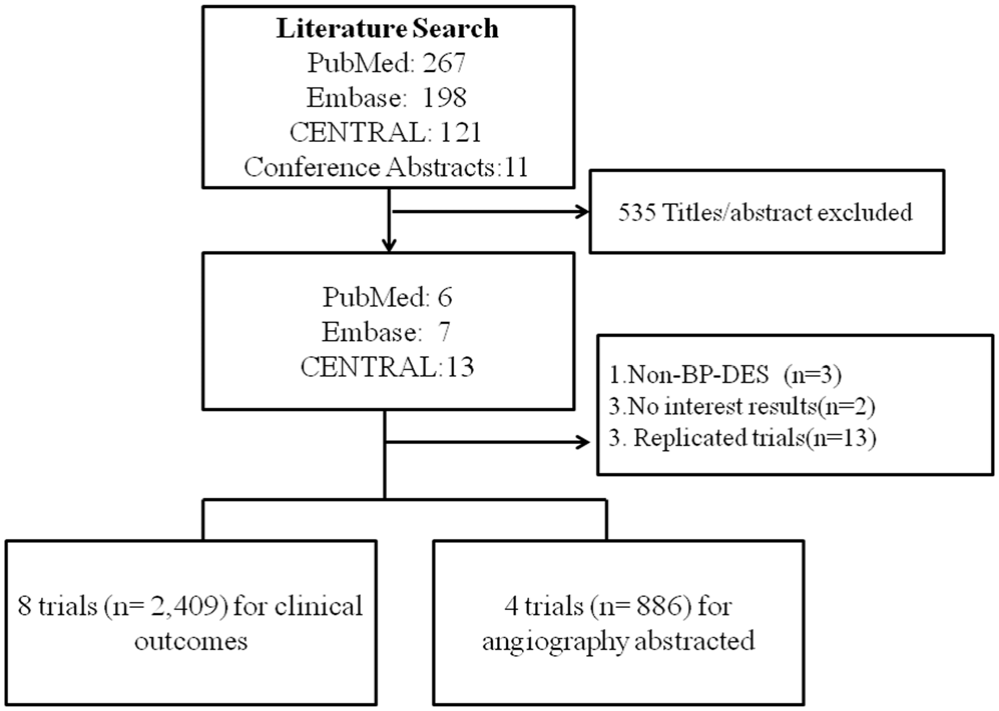
Flow diagram of the review process.

We identified 7 RCTs that satisfied our inclusion criteria (Figure 1). [10], [11], [12], [13], [14], [15], [16], [17] Additional follow-up data on safety and efficacy were available for PAINT trial [11]. The STEALTH trial 5 years and EUCATAX 2 years updated studies are just abstracts without strict peer review and sufficient outcomes data, and therefore excluded [18], [19].

Altogether, 7 trials (n = 2,409) were finally analyzed to compare the clinical outcomes with 1,307 and 1,102 allocated to the BP-DES and BMS, respectively. Four trials were used for angiography evaluation of ISLL. For 3-arm PAINT trial, ISLL data was abstracted to compare BP-DES (sirolimus or paclitaxel arm) to BMS, respectively.

### Baseline Characteristics

The baseline characteristics are described in Table 1. Mean lesion length was 21.01±9.33 mm in the BP-DES group as compared to 20.09±8.84 mm in the BMS group. Mean vessel size was 2.94±0.45 mm in BP-DES and 2.94±0.45 mm in BMS (Table 2). Mean stent size was 3.09±0.38 mm in BP-DES and 3.08±0.63 mm in BMS. Mean stent length was 21.2±7.35 mm in the BP-DES group as compared to 20.39±6.82 mm in the BMS group (Table 3). The target vessel was 33.3% patients with RAD, 30.4% with LCX and 35.8% with LAD in the BP-DES group, as compared to 26.7% patients with RAD, 32.2% with LCX and 40.5% with LAD in the BMS group. Mean age was similar in the two groups (62.9±10.03 vs. 63.1±10.14). Men represented 73.9% of the BP-DES and 77.8% of the BMS population. There were 21.1% patients with diabetes in the BP-DES group and 17.9% in the BMS group. Mean dual anti-platelet duration was similar in the 2 groups (7.3 vs. 6.9 months).

**Table 1:** Main characteristics of the included studies.

No.	Trial	FU (m)	sample		drugs		Male (%)		Age (Year+SD)				DAPT (m)		Diabets%		Admission Diagnosis
			DP	BMS	DP	BMS	DP	BMS	DP		BMS		DP	BMS	DP	BMS	
1	PAINT	36	217	57	P/S	bare	64.1	66.7	59.9	10.4	58.5	9.6	12	12	31.8	26.3	CAD
2	EUROSTAR II	8	152	151	paclitaxel	bare	74.3	68.9	64.9	9.2	66.2	9.4	6	6	26.3	22.5	CAD
3	STEALTH	6	80	40	biolimus	bare	60	82.5	62.2	10.1	61.1	9.4	3	3	26.6	22.5	CAD
4	COMFOR-AMI	12	575	582	biolimus	bare	80.5	78.2	60.7	11.6	60.4	11.9	12	12	14.6	15.5	STIMI
5	EUCATAX	12	211	211	Paclitaxel	bare	83.4	79.1	63.8	10.2	64.7	12.2	6	3	23.2	16.1	CAD
6	CORACTO	24	45	46	sirolimus	bare	69.6	82.2	64.7	9.9	64.8	8.9	6	6	21.7	22.2	CTO
7	FUTURE 1	12	27	15	Everolimus	bare	85.2	86.7	64.2	8.8	65.6	9.6	6	6	3.7	0	CAD

**Table 2:** Vessel Size and Lesion Length of the included studies.

No.	Published	Trial	inclusion	Stent Platform		drugs		Vessel Size (mm+SD)				Lesion Length (mm+SD)			
				DP	BMS	DP	BMS	DP		BMS		DP		BMS	
1	Lemos 2012	PAINT	de novo, native, 2.5–3.5 mm;single stent ≤29 mm	SS	SS	paclitaxel	bare	3.1	0.4	3.1	0.4	NA	NA	NA	NA
				SS	SS	Sirolimus	bare	3.1	0.3	3.1	0.4	21.8	4.8	22.5	5
2	Silber 2011	EUROSTAR II	de novo, native, 2.5–3.5 mm;leision ≤24 mm	CC	SS	paclitaxel	bare	2.74	0.51	2.73	0.48	15.12	7.58	15.16	7.69
3	Grube 2005	STEALTH	de novo, native, 2.75–4.0 mm	SS	SS	biolimus	bare	2.95	0.4	2.97	0.42	15.37	4.64	13.75	3.77
4	Räber 2012	COMFOR-AMI	STIMI	SS	SS	biolimus	bare	3.04	0.47	3.01	0.46	18.19	9.73	17.77	9.57
5	Rodriguez 2011	EUCATAX	de novo, 70% ≤stenosis	SS	SS	Paclitaxel	bare	2.75	0.5	2.85	0.5	16.2	6.1	15.6	6.3
6	Reifart 2010	CORACTO	native, CTO, 2.5–4.5 mm	SS	SS	sirolimus	bare	2.7	0.51	2.8	0.63	39.4	23.1	35.8	20.7
7	Grube 2004	FUTURE 1	de novo, 2.75–4.0 mm;leision ≤18 mm	SS	SS	Everolimus	bare	3.1	0.47	2.96	0.43	NA	NA	NA	NA

**Table 3:** Target Vessel, Stent Length and Stent Diameter of the included studies.

No.	Published			Target Vessel						Stent Length				Stent Diameter			
		drugs		RCA		LCX		LAD		(mm+SD)				(mm+SD)			
		DP	BMS	DP	BMS	DP	BMS	DP	BMS	DP		BMS		DP		BMS	
1	Lemos 2012	paclitaxel	bare	33.3	15.8	44.1	57.9	22.5	26.3	22.5	5.5	22.5	5	3.1	0.4	3.1	0.4
		Sirolimus	bare	25.5	15.8	56.6	57.9	17.9	26.3	21.8	4.8	22.5	5	3.1	0.3	3.1	0.4
2	Silber 2011	paclitaxel	bare	36	31.3	23.8	27	39	40.5	16.98	6.74	17.01	8.29	NA	NA	NA	NA
3	Grube 2005	biolimus	bare	34.1	27.5	37.8	30	28	42.5	19.03	8.76	16.23	5.53	NA	NA	NA	NA
4	Räber 2012	biolimus	bare	45.9	44.6	14.3	15.5	39.3	39.6	25.2	12.7	24.1	12.3	3.2	0.4	3.2	1.1
5	Rodriguez 2011	Paclitaxel	bare	17.6	25.1	18.5	23.8	62.8	48.5	21.7	5.6	20	4.8	2.96	0.4	2.93	0.6
6	Reifart 2010	sirolimus	bare	NA	NA	NA	NA	NA	NA	NA	NA	NA	NA	NA	NA	NA	NA
7	Grube 2004	Everolimus	bare	41	27	18	13	41	60	NA	NA	NA	NA	NA	NA	NA	NA

### Safety Endpoints

#### Death

**Figure 2:**
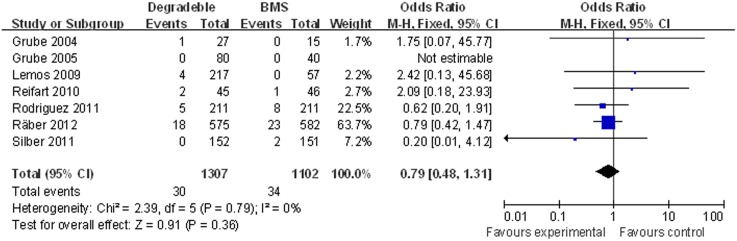
Individual and summary odds ratios for death in patients treated with BP-DES vs. BMS.

There was no significant difference in the rate of death with BP-DES as compared with BMS: 2.29% (30/1,307) in the BP-DES group and 3.09% (34 of 1,102) in the BMS group (OR [95% CI] = 0.79 [0.48–1.31]) (Figure 2).

#### Myocardial infarction

**Figure 3:**
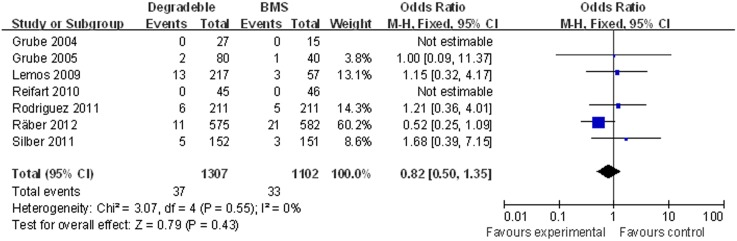
Individual and summary odds ratios for myocardial infarction in patients treated with BP-DES vs. BMS.

There was no significant difference in the rate of MI with BP-DES as compared with BMS: 2.83% (37/1,307) in the BP-DES group and 2.99% (33/1,102) in the BMS group (OR [95% CI] = 0.82 [050–1.35]) (Figure 3).

#### Definite stent thrombosis

**Figure 4:**
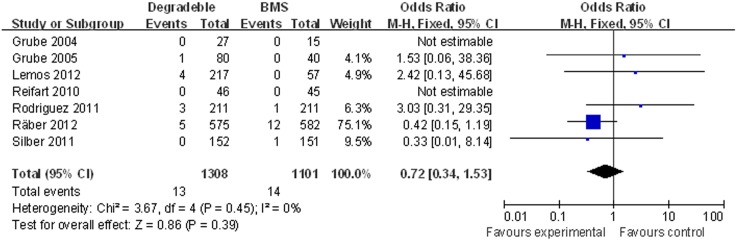
Individual and summary odds ratios for definite stent thrombosis (DST) in patients treated with BP-DES vs. BMS.

Seven studies (2,409 patients) with mean follow-up 10.5 months were included to compare the ST between BP-DES vs. BMS. There was no significant difference in the rate of total DST with BP-DES as compared with BMS: 0.99% (13/1,308) in the BP-DES group and 1.27% (14/1,101) in the BMS group (OR [95% CI] = 0.72 [0.34–1.53]) (Figure 4).

The meta-analysis did not showed a significant decreased late DST in patients treated with BP-DES (0.38%, 5/1,308) as compared to patients receiving BMS (0.18%, 2/1,101) (OR [95% CI] = 0.57 [0.23–1.40]) (Figures S1).

There was no significant difference in the rate of early DST with BP-DES as compared with BMS: 0.54% (7/1,308) in the BP-DES group and 1.59% (12/1,101) in the BMS group (OR [95% CI] = 1.19 [0.30–4.72]) (Figures S2).

### Efficacy Endpoints

#### Target lesion revascularisation

**Figure 5:**
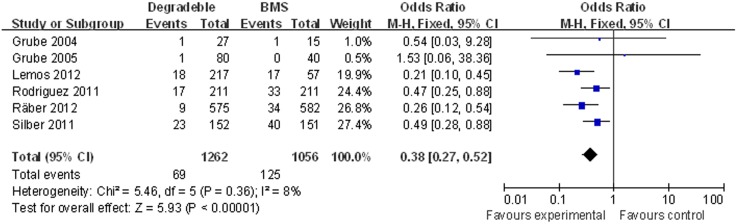
Individual and summary odds ratios for TLR in patients treated with BP-DES vs. BMS.

Six studies with 2,318 patients were included. The meta-analysis showed a significant decreased TLR in patients treated with BP-DES (5.47%, 69/1,262) as compared to patients receiving BMS (11.84%, 125/1,056) (OR [95% CI] = 0.38 [0.27–0.52]) (Figure 5).

#### Target vessel revascularisation

**Figure 6:**
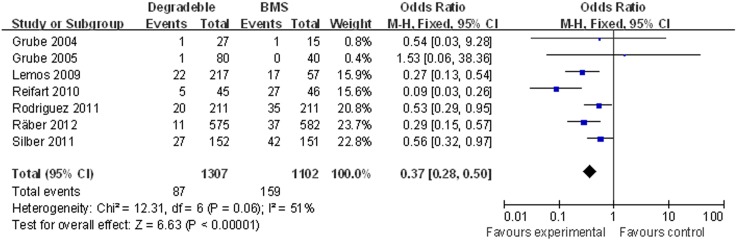
Individual and summary odds ratios for TVR in patients treated with BP-DES vs. BMS.

Seven studies (2,409 patients) with mean follow-up 10.5 months were included. The meta-analysis showed a significant decreased TVR in patients treated with BP-DES (6.66%, 87/1,307) as compared to patients receiving BMS (14.43%, 159/1,102) (OR [95% CI] = 0.37 [0.28–0.50]) (Figure 6).

#### In-stent late loss

**Figure 7:**
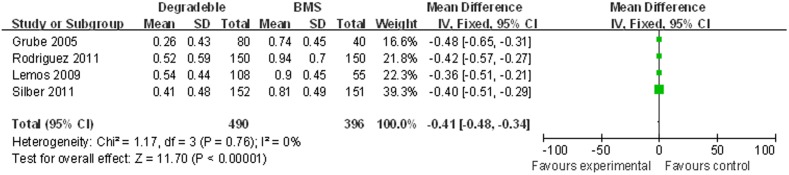
Standardized mean difference (SMD) for ISLL in patients treated with BP-DES vs. BMS.

We included 886 patients with mean follow-up 7 months. ISLL significantly decreased in BP-DES group (0.43±0.49 mm) compared to BMS group (0.85 mm±0.52). (SMD [95% CI] = −0.41 [–0.48; –0.34]), when paclitaxel arm data of PAINT trial was used as BP-DES group. (Figure 7).

The results were confirmed when sirolimus arm data of PAINT trial was used as BP-DES group (0.38±0.48 mm), comparing to BMS group (0.85 mm±0.52). (SMD [95% CI] = −0.46 [–0.53; –0.39]). (Figures S3).

#### Sensitivity and subgroup analyses

Sensitivity analysis was performed by removing each of the studies one at a time, which did not detected any influence of any single study on the overall results.

With regard to ISLL, the overall results in favor of BP-DES were confirmed when paclitaxel-eluting BP-DES were analyzed separately: BP-DES group (0.49±0.50 mm) compared to BMS group (0.88 mm±0.55). (SMD [95% CI] = –0.39 [–0.47; –0.32]). (Figures S4).

Subgroup analysis of outcomes between BMS and BP-BES, as well as biodegradable limus- and sirolimus eluting stents are performed, which confirmed that BP-DES is more effective in reducing ISLL, TVR and TLR, as safe as standard BMS with regard to death, ST and MI. (Figures S5–S21).

## Discussion

This is the first meta-analysis that directly compared outcomes between BP-DES and BMS. The main finding is that patients allocated to BP-DES showed significantly less ISLL, TVR and TLR, with comparable MI, death, DST to those treated with BMS.

Drug-eluting stents (DES) with durable polymer coating rapidly transformed the practice of percutaneous coronary intervention (PCI), by significantly reducing rates of restenosis in comparison with bare-metal stents (BMS). [20], [21] However, residual polymer in the coronary milieu induces inflammatory response at the vessel-wall and then contributes to late thrombotic stent as well as late neointimal overgrowth [22], [23].

Degradable-polymer DES has been developed by providing similar controlled drug release with subsequent degradation of the polymer in 3–9 months and, therefore, appears to be a promising solution to overcome this problem [4], [5]. However, at least 2 benchmarks, efficacy and safety, should be considered when appraising the results of BP-DES. First, they should demonstrate comparable, if not superior, safety results compared with BMS and DES. Second, the BP-DES should also reduce the incidence of revascularization compared with BMS, and be shown to be at least noninferior in regard to contemporary DES [6]. At present, clinical data have accumulated to support the use of biodegradable polymer stents to be a safe and efficacious alternative to conventional durable polymer DES [24], [25].

Several RCTs have been investigated to compare outcomes of BP-DES vs. BMS. FUTURE I was the first prospective, single-blind, randomized trial to evaluate the safety and efficacy of everolimus-eluting stents (EES), coated with a bio-absorbable polymer, comparing with BMS [17]. In this initial clinical experience, BP-DES demonstrated a safe and efficacious method to reduce in-stent neointimal hyperplasia and restenosis. In PAINT trial, Lemos et al tested 2 novel DES, covered with a biodegradable-polymer carrier and releasing paclitaxel or sirolimus, which were compared against a bare metal stent [10]. They found both BD-DES were effective in reducing neointimal hyperplasia and 1-year re-intervention, compared to BMS. The COMFORTABLE AMI [14] is the largest RCTs (1161 patients) to date, comparing outcomes of BP-DES vs. BMS. This study showed that the use of biolimus-eluting stents with a biodegradable polymer resulted in a lower rate of the composite of major adverse cardiac events at 1 year among patients with ST-elevation myocardial infarction undergoing primary PCI. However, all those trials comparing BP-DES vs. BMS were not powerful to reveal potential differences in low frequency events including MI, death and ST, and therefore their relative efficacy and safety remains undetermined.

The findings of the current study are novel and important for at least 2 reasons.

First, our study directly addressed the comparison of outcomes between BP-DES and BMS. We didn’t show a significant difference in death, DST and MI for BP-DES, as compared to BMS. These results should be explained carefully: 1. It is our opinion that an analysis with 2,409 patients was unable to detect a significant advantage may be interpreted as a limited difference between BP-DES and BMS. 2. The low incidence of death, DST and MI has made investigating the difference of these outcomes difficult. Further large RCTs with long-term follow-up are warranted to better define the relative merits of BP-DES.

Recently, 2 large scale network studies have showed BP-DES were associated with significantly lower rates of cardiac death/MI, MI, and ST than BMS [24], [25]. In our opinion, these different results should be noted and could be explained with following reasons: 1. Network analysis is a different statistic method with our meta-analysis, which allows for indirect comparisons of stents not in any of the individual trials (comparison of stent A vs. C by using trials comparing A vs. B and B vs. C), and may include more trials. 2. We included 7 trials of direct comparison of BP-DES vs. BMS for data abstraction. However, Palmerini et al [25] only included 1 trial (COMFORTABLE AMI) in their analysis, indicating that most of their results about BP-DES vs. BMS are based on indirect comparison. 3. Duration of antiplatelet therapy in patients treated with BD-DES and BMS differed across trials and therefore represented a confounding factor in these 2 network analysis.

Secondary, we for the first time reported an improved anti-restenotic efficacy of BP-DES vs. BMS with lower ISLL in 8 months, as well as a significant reduction of BP-DES in both TLR and TVR. This finding is supported by the results of previous network meta-analysis regarding to TVR [24], [25]. Thus, similar findings with different trials further clarified the efficacy profile of BP-DES, as compare to BMS.

DES had revolutionized the practice of interventional cardiology and been implanted in the majority of PCI procedures over the past decades. However, BMS are still used especially in patients with AMI, high bleeding risk or large coronary vessel (>3.0 mm). Thus, the findings of a reduced ISLL, TVR and TLR, as well as non-inferior safety outcomes with BP-DES as compared to BMS in our meta-analysis are clinically significant and indicating a safe and efficacious alternative to conventional BMS.

Multiple studies have shown improved safety and efficacy of second generation everolimus-eluting stent than early-generation sirolimus-eluting, paclitaxel-eluting stents, [26], [27], [28] and therefore, representing the standard care to which new stent designs should be compared. [29] Also, previous studies have also proved superior outcomes with EES when compared to BMS. [30], [31], [32] However, there is only FUTURE I trial comparing the safety and efficacy of biodegradable polymer everolimus-eluting stents (BP-EES) and BMS. This study indicated that BP-EES with biodegradable polymer could be a safe and efficacious method to reduce in-stent neointimal hyperplasia and restenosis. [17].

Biolimus is the limus analogue with the highest lipophilicity used for drug elution on currently available stent platforms. [33] Theoretically, the increased lipophilicity of the drug biolimus may provide a more rapid and homogeneous drug distribution, potentially leading to a more potent anti-inflammatory and antithrombotic local effect. In fact, previous study reported that BP-BES, as compared to PP-EES, showed similar stent coverage and apposition as assessed by OCT at 6–8 months. [34] Furthermore, meta-analysis and clinical trials proved that BP-BES are as safe and efficacious as the current standard of a thin-strut EES with a durable biocompatible polymer. [35], [36], [37], [38] So, BP-BES may also be an alternative standard choice for comparing the stents safety and efficacy.

We compared the outcomes of BMS with BP-BES, as well as biodegradable limus- and sirolimus eluting stents. All the subgroup analysis support our conclusions that BP-DES is more effective in reducing ISLL, TVR and TLR, as safe as standard BMS with regard to death, ST and MI.

## Limitations

1. The limitations of the meta-analytical approach are well known and documented. [39] 2. We didn’t have data for all trials at each time period; therefore, this limited comparison of rates across time within a specific end point. 3. Inclusion criteria were not equivalent across the included trials, however, reflects the broadly inclusive nature of the included patient population. 4. Our meta-analysis might be un-powerful to detect the difference of low incidence events such as MI, death and ST. 5. A major limitation is absence of comparisons with DES like everolimus eluting ones, which represent the standard of care for PCI.

## Conclusions

BP-DES is more effective in reducing ISLL, TVR and TLR, as well as comparable with BMS in regard to death, ST and MI. Further large RCTs with long-term follow-up are warranted to better define the relative merits of BP-DES.

## Supporting Information

Figure S1 Individual and summary odds ratios for late definite stent thrombosis (DST) in patients treated with BP-DES vs. BMS.
(JPG)

Figure S2 Individual and summary odds ratios for early definite stent thrombosis (DST) in patients treated with BP-DES vs. BMS.
(JPG)

Figure S3 Standardized mean difference (SMD) for ISLL in patients treated with BP-DES vs. BMS.
(JPG)

Figure S4 Standardized mean difference (SMD) for ISLL in patients treated with paclitaxel-eluting BP-DES vs. BMS.
(JPG)

Figure S5 Individual and summary odds ratios for death in patients treated with BP-BES vs. BMS.
(JPG)

Figure S6 Individual and summary odds ratios for myocardial infarction in patients treated with BP-BES vs. BMS.
(JPG)

Figure S7 Individual and summary odds ratios for TLR in patients treated with BP-BES vs. BMS.
(JPG)

Figure S8 Individual and summary odds ratios for TVR in patients treated with BP-BES vs. BMS.
(JPG)

Figure S9 Individual and summary odds ratios for early definite stent thrombosis in patients treated with BP-BES vs. BMS.
(JPG)

Figure S10 Individual and summary odds ratios for late definite stent thrombosis (DST) in patients treated with BP-BES vs. BMS.
(JPG)

Figure S11 Individual and summary odds ratios for death in patients treated with BP-limus eluting stents vs. BMS.
(JPG)

Figure S12 Individual and summary odds ratios for myocardial infarction in patients treated with BP-limus eluting stents vs. BMS.
(JPG)

Figure S13 Individual and summary odds ratios for TLR in patients treated with BP-limus eluting stents vs. BMS.
(JPG)

Figure S14 Individual and summary odds ratios for TVR in patients treated with BP-limus eluting stents vs. BMS.
(JPG)

Figure S15 Individual and summary odds ratios for early definite stent thrombosis in patients treated with BP-limus eluting stents vs. BMS.
(JPG)

Figure S16 Individual and summary odds ratios for late definite stent thrombosis in patients treated with BP-limus eluting stents vs. BMS.
(JPG)

Figure S17 Standardized mean difference (SMD) for ISLL in patients treated with BP-limus eluting stents vs. BMS.
(JPG)

Figure S18 Individual and summary odds ratios for death in patients treated with BP-SES vs. BMS.
(JPG)

Figure S19 Individual and summary odds ratios for myocardial infarction in patients treated with BP-SES vs. BMS.
(JPG)

Figure S20 Individual and summary odds ratios for TVR in patients treated with BP-SES vs. BMS.
(JPG)

Figure S21 Individual and summary odds ratios for late define stent thrombosis in patients treated with BP-SES vs. BMS.
(JPG)

Table S1 Protocols of dual anti-platelet therapy (DAPT).
(DOCX)

Checklist S1 PRISMA 2009 Checklist.
(DOC)
