# Supplementary figures and images for: Safety and Efficacy of Biodegradable Drug-Eluting vs. Bare Metal Stents: A Meta-Analysis from Randomized Trials
Source: PLoS One. 2014 Jun 19;9(6):e99648. doi: 10.1371/journal.pone.0099648 (PMC4063774; doi:10.1371/journal.pone.0099648)

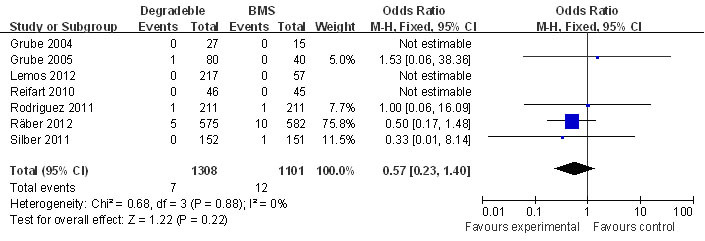

Supplement: Figure S1 — Individual and summary odds ratios for late definite stent thrombosis (DST) in patients treated with BP-DES vs. BMS. (JPG) [file pone.0099648.s001.jpg]

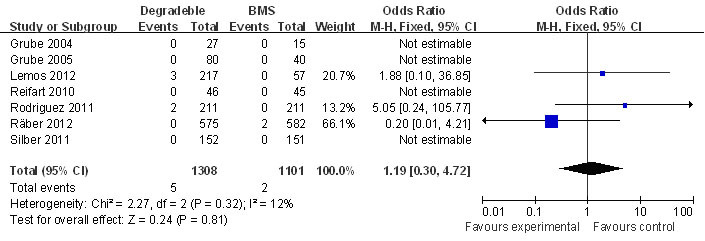

Supplement: Figure S2 — Individual and summary odds ratios for early definite stent thrombosis (DST) in patients treated with BP-DES vs. BMS. (JPG) [file pone.0099648.s002.jpg]

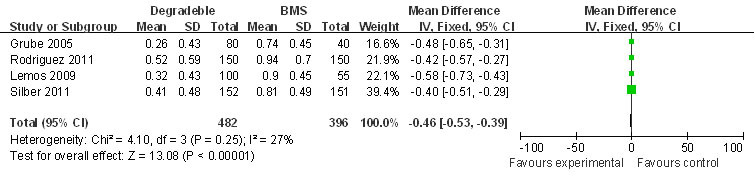

Supplement: Figure S3 — Standardized mean difference (SMD) for ISLL in patients treated with BP-DES vs. BMS. (JPG) [file pone.0099648.s003.jpg]

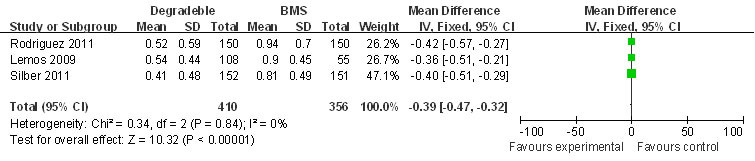

Supplement: Figure S4 — Standardized mean difference (SMD) for ISLL in patients treated with paclitaxel-eluting BP-DES vs. BMS. (JPG) [file pone.0099648.s004.jpg]

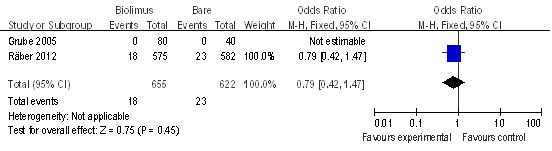

Supplement: Figure S5 — Individual and summary odds ratios for death in patients treated with BP-BES vs. BMS. (JPG) [file pone.0099648.s005.jpg]

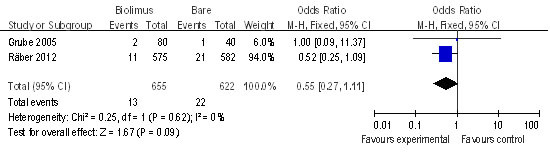

Supplement: Figure S6 — Individual and summary odds ratios for myocardial infarction in patients treated with BP-BES vs. BMS. (JPG) [file pone.0099648.s006.jpg]

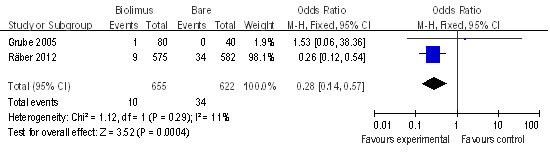

Supplement: Figure S7 — Individual and summary odds ratios for TLR in patients treated with BP-BES vs. BMS. (JPG) [file pone.0099648.s007.jpg]

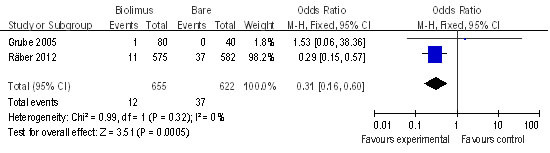

Supplement: Figure S8 — Individual and summary odds ratios for TVR in patients treated with BP-BES vs. BMS. (JPG) [file pone.0099648.s008.jpg]

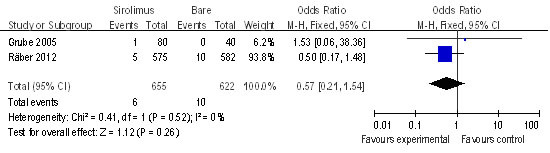

Supplement: Figure S9 — Individual and summary odds ratios for early definite stent thrombosis in patients treated with BP-BES vs. BMS. (JPG) [file pone.0099648.s009.jpg]

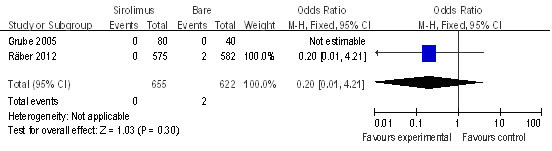

Supplement: Figure S10 — Individual and summary odds ratios for late definite stent thrombosis (DST) in patients treated with BP-BES vs. BMS. (JPG) [file pone.0099648.s010.jpg]

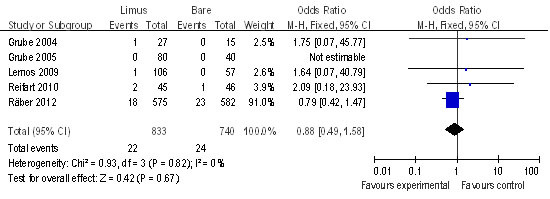

Supplement: Figure S11 — Individual and summary odds ratios for death in patients treated with BP-limus eluting stents vs. BMS. (JPG) [file pone.0099648.s011.jpg]

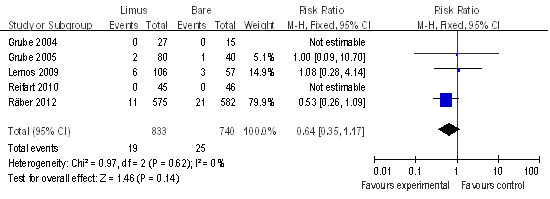

Supplement: Figure S12 — Individual and summary odds ratios for myocardial infarction in patients treated with BP-limus eluting stents vs. BMS. (JPG) [file pone.0099648.s012.jpg]

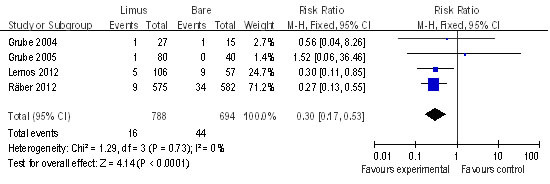

Supplement: Figure S13 — Individual and summary odds ratios for TLR in patients treated with BP-limus eluting stents vs. BMS. (JPG) [file pone.0099648.s013.jpg]

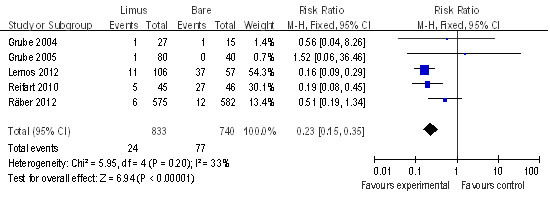

Supplement: Figure S14 — Individual and summary odds ratios for TVR in patients treated with BP-limus eluting stents vs. BMS. (JPG) [file pone.0099648.s014.jpg]

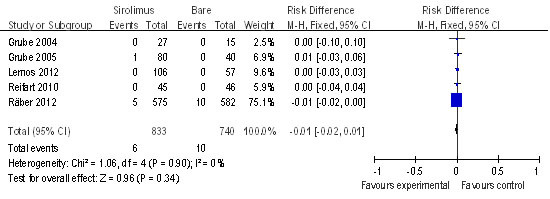

Supplement: Figure S15 — Individual and summary odds ratios for early definite stent thrombosis in patients treated with BP-limus eluting stents vs. BMS. (JPG) [file pone.0099648.s015.jpg]

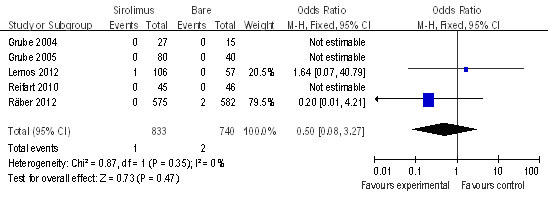

Supplement: Figure S16 — Individual and summary odds ratios for late definite stent thrombosis in patients treated with BP-limus eluting stents vs. BMS. (JPG) [file pone.0099648.s016.jpg]

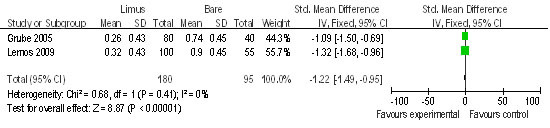

Supplement: Figure S17 — Standardized mean difference (SMD) for ISLL in patients treated with BP-limus eluting stents vs. BMS. (JPG) [file pone.0099648.s017.jpg]

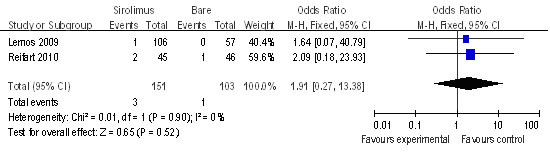

Supplement: Figure S18 — Individual and summary odds ratios for death in patients treated with BP-SES vs. BMS. (JPG) [file pone.0099648.s018.jpg]

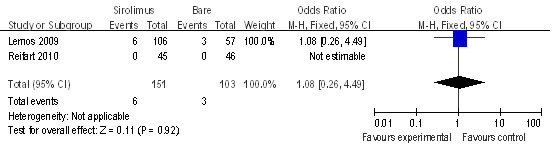

Supplement: Figure S19 — Individual and summary odds ratios for myocardial infarction in patients treated with BP-SES vs. BMS. (JPG) [file pone.0099648.s019.jpg]

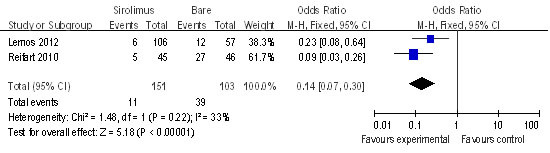

Supplement: Figure S20 — Individual and summary odds ratios for TVR in patients treated with BP-SES vs. BMS. (JPG) [file pone.0099648.s020.jpg]

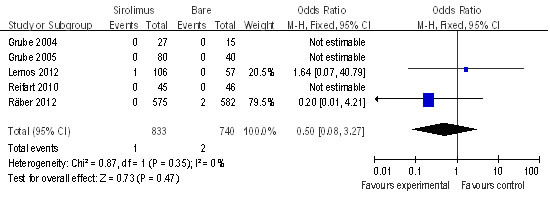

Supplement: Figure S21 — Individual and summary odds ratios for late define stent thrombosis in patients treated with BP-SES vs. BMS. (JPG) [file pone.0099648.s021.jpg]
