# Supplementary material for: Safety and Efficacy of Biodegradable Drug-Eluting vs. Bare Metal Stents: A Meta-Analysis from Randomized Trials
Source: PLoS One. 2014 Jun 19;9(6):e99648. doi: 10.1371/journal.pone.0099648 (PMC4063774; doi:10.1371/journal.pone.0099648)
Supplement: Table S1 — Protocols of dual anti-platelet therapy (DAPT). (DOCX) [file pone.0099648.s022.docx]

Table S1. Protocols of dual anti-platelet therapy(DAPT)

| No. | Published | Trial | Regimes  DP/BMS | | Prior intervention  DP/BMS | After intervention  DP/BMS | DAPT^†^  months | |
| --- | --- | --- | --- | --- | --- | --- | --- | --- |
|  |  |  |  |  |  |  | DP | BMS |
| 1 | Lemos 2012 | PAINT | A^*^+C^#^ | | 80-325 mg A+75-300mg C | 80-325mg/day A+75 mg/day C | 6-12 | 1 |
| 2 | Silber 2005 | EUROSTAR II | A+C |  | 100 mg A +300 mg C | 100mg/day A+75 mg/day C | 6 | 6 |
| 3 | Grube 2011 | STEALTH | A+C or A+T^&^ |  | 300 mg A+300mg C or 500mg T | 250mg/day A+75 mg/day C^@^ | 3 | 3 |
| 4 | Räber 2012 | COMFORTABLE-AMI | A+C or P^$^ |  | 250mg A+600 mg C or 60mg P | 250 mg/day A+75 mg/day C | 12 | 12 |
| 5 | Rodriguez 2011 | EUCATAX | A+C |  | 325mg A+300 mg C | 300 mg/day A+75 mg/day C | 3 | 6 |
| 6 | Reifart 2010 | CORACTO | A+C |  | 500 mg A or 300mg C | 100 mg/day A+75 mg/day C | 6 | 6 |
| 7 | Grube 2004 | FUTURE 1 | A+C |  | 325mg A+300 mg C | 325mg/day A+75 mg/day C | 6 | 6 |

^*^A: aspirin; ^#^ C: clopidogrel; ^$^DAPT: dual anti-platelet therapy; ^&^ T: Ticlopidine; ^@^ or 500mg T for 2 weeks; ^§^P: prasugrel.
